# Effect of Substituting Fish Oil with Camelina Oil on Growth Performance, Fatty Acid Profile, Digestibility, Liver Histology, and Antioxidative Status of Red Seabream (*Pagrus major*)

**DOI:** 10.3390/ani11071990

**Published:** 2021-07-02

**Authors:** Kumbukani Mzengereza, Manabu Ishikawa, Shunsuke Koshio, Saichiro Yokoyama, Zhang Yukun, Ronick S. Shadrack, Seok Seo, Tran Nguyen Duy Khoa, Amina Moss, Serge Dossou, Mohammed Fouad El Basuini, Mahmoud A. O. Dawood

**Affiliations:** 1 The United Graduate School of Agriculture Sciences, Kagoshima University, 1-21-24 Korimoto, Kagoshima 890-0056, Japan; kumbumzenge@gmail.com (K.M.); zhongcy@ffrc.cn (Z.Y.); rspenly@gmail.com (R.S.S.); seoseok91@naver.com (S.S.); 2 Laboratory of Aquatic Animal Nutrition, Faculty of Fisheries, Kagoshima University, Kagoshima 890-0056, Japan; ishikawa@fish.kagoshima-u.ac.jp (M.I.); koshio@fish.kagoshima-u.ac.jp (S.K.); yokoyama@fish.kagoshima-u.ac.jp (S.Y.); 3 Department of Aquatic and Fisheries Science, Private Bag 201, Mzuzu University, Mzuzu 105200, Malawi; 4 Laboratory of Larval Rearing Management, Faculty of Fisheries, Kagoshima University, Kagoshima 890-0056, Japan; tndkhoa@ctu.edu.vn; 5 College of Aquaculture and Fisheries, Can Tho University, Campus ll, Nihn Kieu District, Can Tho City 9000, Vietnam; 6 Faculty of Pure and Applied Sciences, University of The Bahamas, University Drive, Nassau N-4912, Bahamas; aminasmoss@gmail.com; 7 Laboratoire d’Hydrobiologie et Aquaculture, Faculté des Sciences Agronomies’, Université d’Abomey Calavi, Cotonou 01 BP 526, Benin; sergedos@yahoo.fr; 8 Faculty of Desert Agriculture, King Salman International University, South Sinai 46612, Egypt; m_fouad_islam@yahoo.com; 9 Department of Animal Production, Faculty of Agriculture, Tanta University, Tanta 31527, Egypt; 10 Department of Animal Production, Faculty of Agriculture, Kafrelsheikh University, Kafr El-Sheikh 33516, Egypt

**Keywords:** camelina oil, liver histology, oxidative status, red seabream

## Abstract

**Simple Summary:**

The shortage of natural resources, prices, and high demand for fish oil has encouraged the use of non-traditional ingredients in aquafeed. The search for an alternative lipid source in aquafeeds has seen terrestrial vegetable oils at the epicenter of various flagship aqua-feed research. Herein, we investigated the effects of substituting fish oil (FO) with camelina oil (CO) on growth performance, fatty acid profile, digestibility, liver histology, and antioxidative status of red seabream (*Pagrus major*). After 56 days of the feeding trial, the results suggested that FO can be replaced with CO in the feeds of farmed red seabream without compromising growth, blood chemistry, digestibility, and overall health status.

**Abstract:**

A 56-day feeding trial to evaluate the responses of red seabream (initial weight: 1.8 ± 0.02 g) to the substitution of fish oil (FO) with camelina oil (CO) at different ratios was conducted. The control diet formulated at 46% CP (6F0C) contained only FO without CO; from the second to the fifth diet, the FO was substituted with CO at rates of 5:1 (5F1C), 4:2 (4F2C), 3:3 (3F3C), 2:4 (2F4C), and 0:6 (0F6C). The results of the present study showed that up to full substitution of FO with CO showed no significant effect on growth variables BW = 26.2 g–28.3 g), body weight gain (BWG = 1275.5–1365.3%), specific growth rate (SGR = 4.6–4.7), feed intake (FI = 25.6–27.8), feed conversion ratio (FCR = 1.0–1.1), biometric indices condition factor (CF = 2.2–2.4), hepatosomatic index (HSI = 0.9–1.1), viscerasomatic index (VSI = 7.5–9.5), and survival rates (SR = 82.2–100) with different FO substitution levels with CO. Similarly, there were no significant differences (*p* < 0.05) found in the whole-body composition except for the crude lipid content, and the highest value was observed in the control group (291 g/kg) compared to the other groups FO5CO1 (232 k/kg), FO4CO2 (212 g/kg), FO2CO4 (232 g/kg) and FO0CO6 (244 g/kg). Blood chemistry levels were not influenced in response to test diets: hematocrit (36–33%), glucose (Glu = 78.3–71.3 mg/dL), total protein (T-pro = 3.1–3.8 g/dL), total cholesterol (T-Chol = 196.0–241 mg/dL), blood urea nitrogen (BUN = 9.0–14.6 mg/dL), total bilirubin (T-Bil = 0.4–0.5 mg/dL), triglyceride (TG = 393.3–497.6 mg/dL), alanine aminotransferase test (ALT = 50–65.5 UL/L), aspartate aminotransferase test (AST = 38–69.3 UL/L). A remarkable modulation was observed in catalase (CAT) and superoxide dismutase (SOD) activities in the liver, as CAT and SOD values were lower with the complete FO substitution with CO (0F6C), and the highest values were observed in the control and (4F2C). This study indicates that red seabream may have the ability to maintain LC-PUFAs between tissues and diets, and CO substitution of FO could improve both lipid metabolism and oxidation resistance as well as maintain digestibility. In conclusion, dietary FO can be replaced up to 100% or 95% by CO in the diets of red seabream as long as n-3 HUFA, EPA, and DHA are incorporated at the recommended level.

## 1. Introduction

Highly unsaturated fatty acids (HUFAs), such as eicosapentaenoic acid (EPA) and docosahexaenoic acid (DHA) are certified components of human health with neurological, immunomodulatory, pathological, cardiovascular, and carcinogenic benefits [1]. Fish are a major source of EPA and DHA [2]. Globally, aquaculture is grappling with a low supply of fish oil (FO) as the main lipid source for fish feed [3,4]. To augment FO fluctuations, the search for an alternative lipid source in aquafeeds has seen terrestrial vegetable oils at the epicenter of various flagship aqua-feed research [5].

Dietary de novo oilseed *Camelina sativa* (L. Crantz) is an ancient crop that originated in Germany around 600 B.C. and is cultivated traditionally in central Europe as an oil crop [6]. Camelina production plummeted in the Middle Ages (5th to 15th centuries), but still evolved as a weed with flax, and has in modern days been coined the “false flax.” Camelina is a member of the Cruciferae (Brassicaceae) family, together with mustards, rapes, canola, radish, turnip, broccoli, cabbage, collards, cauliflower, rutabaga, Brussels sprouts, kohlrabi, and many weeds [7]. Utilization of camelina oil (CO) has been reported in various carnivorous fish species; up to 100 % of added dietary FO was replaced by CO without adverse effects on growth performance, nutrient utilization, and proximate carcass composition of rainbow trout (*Oncorhynchus mykiss*) [8]. In addition, Huyben, et al. [9] demonstrated that up to 40% of FO can be replaced with CO without negative effects on growth performance, fillet fatty acid profile and gut microbiome of gilthead sea bream (*Sparus aurata*).

Elsewhere, supplementation of genetically modified (GM) camelina oil diets of European sea bass (*Dicentrarchus labrax* L.) triggered a metabolic up regulation of both β-oxidation (cpt1a) and fatty acid transport (fabp1) [10]. The authors opinioned that GM camelina oil is an excellent source of EPA and DHA and thus an ideal substitute for FO in diets of marine carnivorous species, contributing to bridging the gap between supply and demand for n-3 LC PUFA while also maintaining or increasing tissue n-3 LC PUFA contents [10].

Novel camelina oil (CO) contain high contents of 18:2 n-6 (linoleic acid), 18:1 n-9 (oleic acid) omega 3 α-linolenic acid (ALA, C18:3n-3), which is an essential fatty acid for fish [11,12,13,14]. Previous studies have shown that FO substitution by appropriate proportions of CO in the feed maintains fish growth response as well as fish health [12]. Total replacement of FO with camelina oil in diets for tilapia could be a suitable alternative for culture, since the growth performance of fish fed total camelina oil diets was not affected compared to a total FO diet, as well as a typical commercial diet. Fatty acid concentrations were significantly modified after 8 weeks of trial, and although camelina oil not enriched tilapia fillet with EPA + DHA at the level of the FO, it efficiently maintained an n-3/n-6 ratio within the recommendation for the prevention of cardiovascular diseases [15]. Camelina oil is less susceptible to oxidative stress because it contains a high amount of γ-tocopherol, the most potent antioxidant tocopherol isomer [16].

Red seabream (*Pagrus major*), an exclusively carnivorous marine fish, is commonly cultured in Japan and other countries [17,18]. Several works have been performed on the impact of dietary vegetable oil and algal lipid source feeding on red seabream growth responses and fatty acids [19,20,21]. Studies have also reported the effects of vegetable and algal lipid supplementation in diets of red seabream on blood function, immunity, growth response, oxidative status, and nutrient digestibility, but effects of dietary oils on tissue histology of red seabream are limited and sporadic. In fact, research on the utilization of CO in red seabream diets is limited. The objective of our study was to determine the effect of FO replacement with CO on the growth performance, fatty acid profile, blood health, fatty acid digestibility, histology of the liver, and oxidative status of red seabream.

## 2. Materials and Methods

### 2.1. Experimental Diets

Five experimental diets had homogeneous nutrient contents of energy (259.4 kJ/g), crude protein (506.6 g/kg), and lipids (143 g/kg) formulated with different ratios of FO and CO. The control diet (FM: 46%) (6F0C) contained FO without CO, and the other four experimental diets were formulated by gradually substituting FO with CO at rates of 5:1 (5F1C), 4:2 (4F2C), 3:3 (3F3C), 2:4 (2F4C), and 0:6 (0F6C) (Table 1 and Table 2). The main sources of protein in the feed were fishmeal and soybean meal. A blend of soybean lecithin, FO, CO, DHA, and EPA were used as lipid sources. Dextrin was used as a carbohydrate source. Activated gluten was added to the mixture as a binder to improve pellet cohesion and avoid pellet leaching. Dried ingredients were ground, sieved through a uniform mesh to maintain a homogenous size, and mixed in a food mixer for 15 min. The liquid form ingredients were homogenized in a sonicator (CA4455Z, Kaijo us Corporation, Tokyo, Japan) before mixing with dry ingredients. Water was added to the feed ingredients to form a dough that was pelleted (1.2–2.2 mm in diameter) using a mincer (ROYAL Inc., Tokyo, Japan). Feed pellets were dried at 60 °C for 120 min, and the dried pellets were stored in plastic bags at −20 °C until use.

### 2.2. Husbandry

Red seabream juveniles were procured and transported from Tawaki Suisan Ltd., Kumamoto Prefecture, Japan, to Kamoike Marine Production Laboratory, Faculty of Fisheries, Kagoshima University, Japan. Fish were stocked indoors in 500 L polyethylene tanks for a week to acclimatize to laboratory conditions while they were fed a commercial diet (45% crude protein; Higashimaru Foods Ltd., Kagoshima, Japan). At the onset of the feeding trial, 20 fish (1.8 g ± 0.02) per tank (three tanks per treatment) were randomly stocked into fifteen polyethylene tanks with 100 L capacity (filled with 80 L of water) in a flow-through seawater system (2.5 L/min) and continuous aeration under a 12 h light/12 h dark photoperiod regime. Water parameters of rearing tanks through the experimental period were 26.4 ± 1.2 °C, 32.1 ± 0.5 g/L of salinity, 6.31 ± 0.1 mg/L of dissolved oxygen, and 7.4 of pH 70% of the water in culturing tanks was exchanged with new sea water every day to maintain favorable conditions for growth and survival of cultured fish. Fish were manually fed twice daily (08:00 and 16:00 h) until apparent satiation and uneaten diets were collected, dried, and weighted to determine the actual feed intake.

### 2.3. Performance Variables and Biometric Indices

At the end of the feeding trial, the fish weight and length were measured individually. Growth indices, feed utilization, and survival rates were calculated using the following formulae:

(1) $$\mathrm{Weight}\;\mathrm{Gain}\;\left(\mathrm{WG}\;\%\right)=\frac{W_{56d}{\;-\;W}_{0d}}{W_{0d}}\times 100$$

(2) $$\mathrm{Specific}\;\mathrm{Growth}\;\mathrm{Rate}\;\left(\mathrm{SGR}\;\%\;/\mathrm{day}\right)=\frac{{\mathrm{Ln}\;W}_{56d}{\;-\;\mathrm{Ln}\;W}_{0d}}{T}\times 100$$

(3) $$\mathrm{Feed}\;\mathrm{Intake}\;\left(\mathrm{FI},\;g/\mathrm{fish}/56\;d\right)=\frac{\mathrm{Dry}\;\mathrm{diet}\;\mathrm{given}\;-\;\mathrm{Dry}\;\mathrm{uneaten}\;\mathrm{diet}\;\mathrm{recovered}}{\mathrm{No}.\;\mathrm{of}\;\mathrm{fish}}$$

(4) $$\mathrm{Feed}\;\mathrm{Conversion}\;\mathrm{Ratio}\;\left(\mathrm{FCR}\right)=\frac{\mathrm{FI}\;\left(g\right)}{\mathrm{WG}\;\left(g\right)}$$

(5) $$\mathrm{Survival}\;\mathrm{rate}\;\left(\mathrm{SR}\;\%\right)=\frac{N_{56d}}{N_{0d}}\times 100$$

(6) $$\mathrm{Condition}\;\mathrm{factor}\;\left(\mathrm{CF}\right)=\frac{W}{L^{3}}\times 100$$

where W56d = final body weight at 56 days, W0d = initial body weight, T = the experimental period in days (d), N0d = initial number of fish, N56d = final number of fish, W = total fish weight (g), and L = total fish length (cm).

Nine fish were collected randomly per treatment, anesthetized (2-phenoxyethanol, 200 μL/L) and the liver and viscera were eviscerated on the ice surface for hepatosomatic (HSI) and viscerasomatic (VSI) indices. Portions of the collected liver were used for histological studies and hepatic antioxidant analysis.

(7) $$\mathrm{HSI}=\frac{\mathrm{Liver}\;\mathrm{weight},\;g}{\mathrm{Fish}\;\mathrm{body}\;\mathrm{weight},\;g\;}\times 100$$

(8) $$\mathrm{VSI}=\frac{\mathrm{Viscera}\;\mathrm{weight},\;g}{\mathrm{Fish}\;\mathrm{body}\;\mathrm{weight},\;g\;}\times 100$$

### 2.4. Proximate Composition Analysis, Fatty Acid and Digestibility Assessment

Samples of feed and fish (four fish per tank) were used for proximate composition determination according to standard procedures [22]. Briefly, moisture content was obtained after drying in an oven at 135 °C for 5 h. Ash was determined after incineration at 550 °C for 6 h. The crude protein content was obtained by determining the nitrogen content (N × 6.25) using automated Kjeldahl analysis (Tecator Kjeltec Auto 2100 analyzer, Foss, Sweden). Crude lipids were gravimetrically determined using a Soxhlet apparatus.

Total lipids were extracted using a chloroform methanol solution and measured by gravimetry after nitrogen drying. To determine the fatty acid composition of total lipids, fatty acid methyl esters (FAMES) in samples were prepared by transesterification using boron trifluoride in methanol and dichloromethane [23]. To determine the fatty acid composition in diets and tissues, FAMEs were identified by comparison of the equivalent chain length (ECL) value and quantified standards (C23:0 methyl esters) determined by a Shimadzu AOC-20I GC 2010 equipped with a flame ionization detector (Supelco, Inc., Japan), and the chromatogram peak areas of total lipids, 5α-cholestane, and fatty acids in the feed were compared directly to those of total lipid, 5α-cholestane and fatty acids in the feces of fish. Digestibility was calculated using the equation described by Sigurgisladottir, et al. [24].

Dry matter digestibility = 100 − (cholestane in diet / cholestane in faeces) × 100(9)

Nutrient digestibility = 100 − ((cholestane in diet / cholestane in faeces) × (nutrient in diet / cholestane in diet) × 100)(10)

### 2.5. Blood Hematological Parameters

Heparinized disposable syringes (1600 IU/mL) were used to collect blood from 5 fish/tank. The hematocrit was determined using the micro-hematocrit technique. Then, plasma was obtained by centrifuging the blood samples at 3000× *g* for 15 min under 4 °C, and then stored at −20 °C until analyses. Glucose (Glu), total cholesterol (T-Chol), blood urea nitrogen (BUN), total bilirubin (T-Bil), alanine aminotransferase test (ALT) aspartate aminotransferase test (AST), total protein (T-pro), and triglyceride (TG) levels were measured using an automated analyzer (SPOTCHEM EZ model SP-4430, Arkray, Inc. Kyoto, Japan).

### 2.6. Antioxidants Activity

First, liver and muscle samples were homogenized in cold iced 0.86% NaCl solution and centrifuged at 4 °C and 12,000 rpm for 10 min. The supernatant of liver samples and blood plasma were determined using a microplate reader (Multiskan GO; Thermo Fisher Scientific, K. K., Tokyo, Japan). SOD activity was determined using the Kit-WST assay (Dojindo Molecular Technologies, Inc., Rockville, MD, USA) at 450 nm. The catalase activity (CAT) assay was performed using spectrophotometric determination of hydrogen peroxide (H_2_O_2_) which forms a stable complex with ammonium molybdate that absorbs at 405 nm.

### 2.7. Hepatic Histopathological Assessment

Liver samples were cut into small pieces and immersed in Bouin solution for 12 h. The fixed tissues were processed routinely in alcohol and rinsed every 24 h until clear. Tissues were embedded in paraffin blocks, sectioned, deparaffinized, and rehydrated using standard techniques. Sagittal sections (5 μm thickness) were obtained using a rotary microtome (RM 2135, Leica, Nussloch, Germany), placed on glass slides, rehydrated, and stained with hematoxylin and eosin. Finally, the slide was permanently mounted (Entellan, EMD Millipore, Billerica, MA, USA) and examined under a light microscope (BX41, Olympus, Tokyo, Japan).

### 2.8. Statistical Analysis

Data on all parameters were pooled and subjected to verification for normality and homogeneity of variances using Shapiro–Wilk and Levene tests, respectively. One-way analysis of variance (ANOVA) was performed on all data. Significantly different mean data groups were located using Fisher’s least significant differences (LSD) test. All statistical analyses were performed using Super ANOVA 1.11, Abacus Concepts, Berkeley, California, USA. Data sets are presented as mean ± standard error of the mean (SEM, n).

## 3. Results

### 3.1. Growth Performance Variables

Table 3 shows the growth, feed utilization, biometric indices, and survival rates of red seabream fed the experimental diets for 56 days. The average initial body weight of red seabream juveniles was 1.8 g. After the 8-week feeding trial, no significant differences were observed in body weight (BW = 26.2–28.3 g), body weight gain (BWG = 1275.5–1365.3%), specific growth rate (SGR = 4.6–4.7), feed intake (FI = 25.6–27.8), feed conversion ratio (FCR = 1.0–1.1), condition factor (CF = 2.2–2.4), hepatosomatic index (HSI = 0.9–1.1), viscerasomatic index (VSI = 7.5–9.5), and survival rate (SR = 82.2–100) of fish fed with different FO substitution levels with CO.

### 3.2. Proximate Composition of Fish Whole Body

Table 4 shows the proximate composition of the red seabream whole body after 56 d of the feeding period. There was no significant alteration in the composition of the red seabream body, except for the crude lipid content. The basal diet (6F0C = free of CO) showed the highest lipid content (291 g/kg) compared to the other groups FO5CO1 (232 k/kg), FO4CO2 (212 g/kg), FO2CO4 (232 g/kg) and FO0CO6 (244k g/kg).

The contents of EPA, DHA, and Σn-3 PUFA in juvenile red seabream decreased with increasing CO replacement levels, and C18:1n-9, C18:2n-6, and C18:3n-3 PUFA increased with the corresponding increase in FO replacement by CO (Table 5 and Table 6). It is worth highlighting that ARA, EPA, and DHA in the liver and muscle of red seabream were mirror images of those detected in the respective diets. The n-3:n-6 ratio between diets and tissues was constant (Table 5 and Table 6), indicating efficient bioconversion of n-3 PUFA from CO.

### 3.3. Apparent Nutrient Digestibility

Apparent digestibility of the different diets with varying CO inclusion in relation to chain length for red seabream is shown in Table 7. Generally, n-3 fatty acids (95–97%) exhibited higher apparent digestibility, and saturated fatty acids (SFA) showed lower apparent digestibility percentages (88–93%). There was no significant difference (*p* > 0.05) in the apparent digestibility of MUFA among the experimental groups. Fish fed the 5F1C diet showed lower digestibility values of saturated, n-6 (88.1%), and n-3 fatty acids (92.1%), and the highest values were in the 4F2C group (93.6% saturates, 97% MUFA; 97.7 %; n-6 and 99%; n-3).

### 3.4. Blood Chemical Parameter

The hematocrit and plasma chemical parameter results are tabulated in Table 8. Substitution of FO with CO at increasing rates showed no significant differences (*p* < 0.05) in blood composition in terms of hematocrit (36–33%), glucose (Glu = 78.3–71.3 mg/dL), total protein (T-pro = 3.1–3.8 g/dL), total cholesterol (T-chol = 196.0–241 mg/dL), blood urea nitrogen (BUN = 9.0–14.6 mg/dL), total bilirubin (T-Bil = 0.4–0.5 mg/dL), triglyceride (TG = 393.3–497.6 mg/dL), alanine aminotransferase test (ALT = 50–65.5 Ul/L), aspartate aminotransferase test (AST= 38–69.3 Ul/L).

### 3.5. Antioxidants Capacity

**Figure 1:**
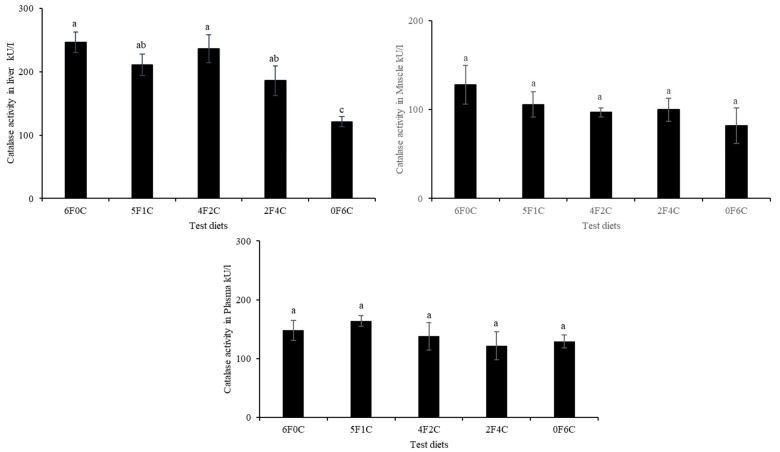
Catalase activities in liver, muscle, and plasma of red seabream after 56 days of the experimental period. Absence of superscript letters refers to non-significant differences between treatments (*p* > 0.05) and presence of different superscript letters refers to significant differences between treatments (*p* < 0.05).

**Figure 2:**
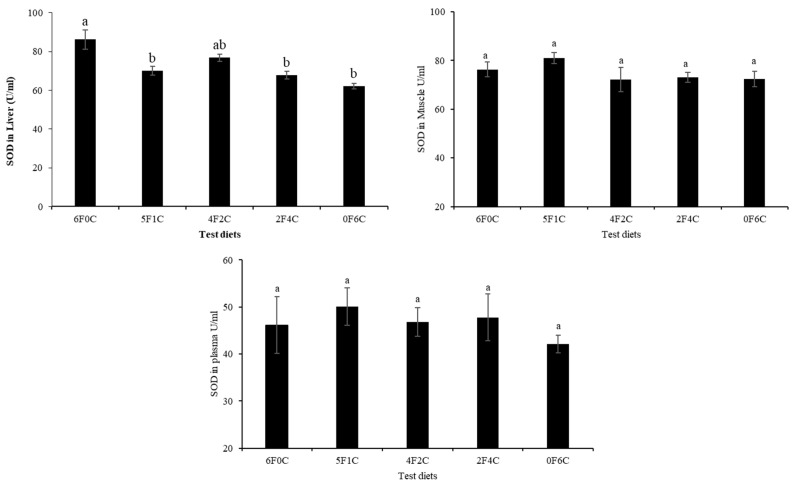
Superoxide dismutase (SOD) activities in liver, muscle, and plasma of red seabream after 56 days of the experimental period. Absence of superscript letters refers to non-significant differences between treatments (*p* > 0.05) and presence of different superscript letters refers to significant differences between treatments (*p* < 0.05).

Figure 1 and Figure 2 show CAT and SOD activities in the liver, muscle, and plasma of red seabream after 56 days of the experimental period. No remarkable alterations in CAT and SOD levels were observed, except in the liver. Replacing FO with CO in a ratio of 4:2 (4F2C) did not cause any difference in the values of the liver CAT and SOD, while a remarkable reduction occurred with the complete substitution of FO with CO (0F6C), and the highest values were observed in the control and (4F2C).

### 3.6. Hepatic Histological Examination

**Figure 3:**
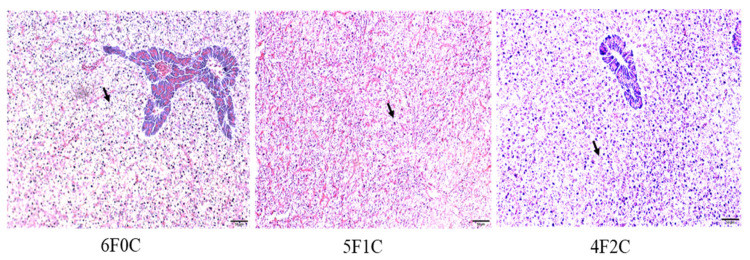

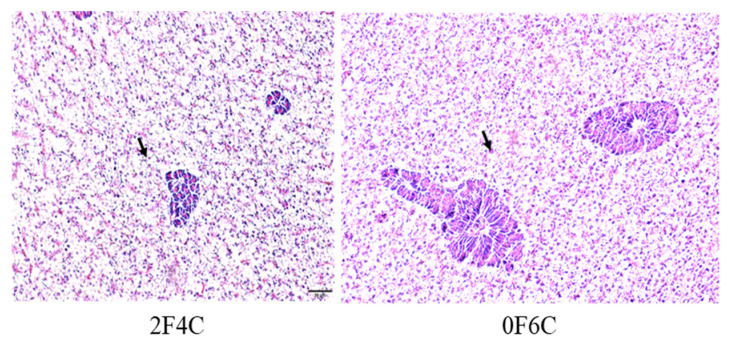
Cross-section in liver of red seabream fed experimental diets for 56 days. Arrows indicate small lipid droplets and lipid vacuoles of the hepatocytes. Hematoxylin and eosin (H&E) Staining, scale = 50 µm).

Cross-sections of the liver of red seabream fed experimental diets for 56 days are shown in Figure 3. Small lipid droplets and lipid vacuoles of the hepatocytes were clear in fish groups fed on 6F0C and 2F4C, and more pronounced in the fish fed the 0F6C diet than in those fed the 5F1C and 4F2C diets.

## 4. Discussion

### 4.1. Growth Performance and Nutrient Utilization

The results of the present trial demonstrated that diets containing CO could completely replace FO without negatively affecting the growth performance and health status of red seabream. Previous studies have shown that FO can be partially substituted by vegetable canola oil (FM: 50%) [25] and palm oil (FM: 67%) [21] in the red seabream diet. Growth performance of red seabream juveniles was gradually impeded by incremental dietary palm oil, and the decline in growth could have been caused by the lowering of EPA and DHA in diets [21]. FO can be completely substituted with vegetable oil to satisfy the requirements of n-3 HUFA [26]. Koshio [26] recommended inclusion of 5–10 g/kg of the diet for limited essential n-3 highly unsaturated fatty acids EPA and DHA, respectively, for juvenile red seabream. The present study is consistent with studies by Betancor, et al. [14] (FM: 49.8%, FM: 25%) on gilthead sea bream and Hixson, et al. [13] (FM: 34.9%, FM: 47.7%) on rainbow trout (*Oncorhynchus mykiss*), which did not show significant differences in growth response to dietary FO full substitution with CO. We attribute the growth maintenance to proportions of EPA and DHA incorporated in diets as well as fish meal, which might have compensated for the low LC-PUFA in CO.

In the present feed trial, the addition of dietary CO did not completely alter the body composition of red seabream. Although the present study showed a slight increase in crude lipid in FO control compared to all other diets, it did not warrant changes in overall red seabream performance and health across all red seabream groups. Similarly, red seabream-fed canola oil diets (FM: 50%) exhibited a uniform chemical proximate composition in all parameters [25]. The fatty acid compositions of vegetable oils and IFO are inherently different. Therefore, substituting FO with vegetable oils inevitably influences ARA, EPA, and DHA [27]. In the present study, the contents of EPA, DHA, and Σn-3 PUFA in juvenile red seabream decreased with increasing CO replacement levels, and C18:1n-9, C18:2n-6, and C18:3n-3 PUFA increased with the corresponding increase in FO replacement by CO. The results reflected the changes in fatty acid profiles of their respective diets to a great extent, indicating that dietary fatty acid profiles strongly influenced fatty acid composition in the muscle and liver of red seabream. It is worth highlighting that ARA, EPA, and DHA in the liver and muscle of red seabream were mirror images of those detected in the respective diets. Thus, our present study has shown that the fatty acid elongase and fatty acid oxidase enzymes in the liver and muscle of juvenile red seabream were not expressed when dietary FO were replaced by CO. We speculate that the almost exclusively similar levels of ARA, EPA, and DHA in diets and tissues indicate that red seabream has limited capacity to biosynthesize DHA and EPA from short chain fatty acids, including ALA. Bell, et al. [28] reasoned that marine fish have a poor ability to synthesize long-chain PUFAs. The n-3:n-6 ratio between diets and tissues was constant, indicating efficient bioconversion of n-3 PUFA from CO. High levels of n-3 LC-PUFA in CO are vital for effective replacement of FO in diets for marine species, such as sea bream, that have limited capacity to endogenously produce DHA and EPA and depend on their inclusion in the diet [9]. Therefore, it is worth noting that CO has an important twin effect as it maintains EPA and DHA levels in the liver and filets, while at the same time fostering a better n-3: n-6 ratio that plays great immunomodulatory and pathological roles in fish and human health. Transgenic camelina oil, at full replacement of FO, was effective substitute for FO as a dietary lipid source of n-3 LC-PUFA in diets for rainbow trout. Fish fed high levels of transgenic camelina oil enriched with EPA and DHA (HCO diet) had FA profiles that were generally similar to those of fish fed FO [29].

### 4.2. Fatty Acid Digestibility

The apparent digestibility estimations (ADC fatty acid) obtained in the present trial were generally high and consistent with those reported previously for red seabream [30] as well as those of other species. CO is highly digestible (95.9%) and is utilized as an energy source by Atlantic salmon [31]. Double bonds are fundamental determinants of the unsaturation of fatty acids. Highly unsaturated fatty acids with more carbon molecules are easily melted and easily diffuse [32]. Therefore, highly unsaturated fatty acids are likely to be easily digested because of their high melting points [33]. In this analysis, n-3 was found to be the most easily digested fatty acid, followed by n-6, saturated fatty acids, and monounsaturated fatty acids, which were the least digestible. Ultimately, the combination degree of unsaturation and the melting point of individual fatty acids resulted in the apparent digestibility of PUFA < MUFA < SFA and short-chain < longer-chain fatty acids, as reported extensively for several species [33]. Our present study shows a slight modification to the previously reported PUFA < SFA < MUFA, but similarly maintains that PUFAs are more easily digested than saturated and mono-unsaturated fatty acids.

### 4.3. Blood Chemistry

Blood parameters are reliable indicators of fish health and are dependent on environmental cues such as temperature, season, and nutritional status [34,35]. The results of the present trial show that all blood parameters and hematocrit mirrored those previously reported for red seabream [35], and there were no statistical differences among different red seabream groups fed different diets.

### 4.4. Lipid Peroxidation

Previous research has shown that dietary ingredients, including vegetable oils, have an influence on lipid peroxidation. The present trial investigated the impact of dietary de novo CO on oxidative stress stability as an important parameter in the nutritional evaluation of FO alternatives. Polyunsaturated fatty acids (PUFAs) in aquafeeds, particularly EPA and DHA, are highly susceptible to reactive oxygen species (ROS) and reactive nitrogen species (RNS), which form peroxides and free radicals [36]. Peroxidation is deleterious because it damages tissue cells and subsequently alters physiological and biochemical processes. The first line of the oxidative defense mechanism is enzymatic, including catalase (CAT), superoxide dismutase (SOD), glutathione reductase (GR), and glutathione peroxidase (GPx), as well as low molecular weight substances such as thiorbaburtic acid reactive substances (TBARS), vitamins (C and E), glutathione (GSH), bilirubin, and flavonoids [37,38,39].

Oxidative stress occurs when excess free radicals are produced in the body, including ROS and RNS [40,41]. When the oxidation level exceeds the removal of oxides, cells and tissues are damaged [42,43]. SOD and CAT enzymes are fundamental for defense against oxygen radicals, thereby preventing a chain of reactions triggered by superoxide radicals [44,45]. TBARS are non-enzyme low molecules that play a similar role in scavenging free radicals [46,47]. Changes in the activities of these enzymes within the antioxidant system often denote oxidative disturbance that may promote stress and usually orchestrated by surpassed generation of free radicals/ ROS against antioxidants/enzymes functions in the cell or tissues [48]. Additionally, the imbalance (in the antioxidant system) created can trigger oxidative condition, leading to cellular damage and predisposes the organisms to multiple disease conditions [48,49]. The results of the present study show that no remarkable differences in CAT and SOD were observed except for in the liver, as a remarkable reduction occurred with the complete substitution of FO with CO (0F6C), and the highest values were observed in the control and (4F2C). The overarching trend shows that the red seabream oxidation resistance were enhanced after CO substitution of FO. CAT is an essential enzyme in biological defense systems. However, the levels of CAT in this experiment were not significantly different [50,51]. The results of the present study correspond with earlier observations by Long, et al. [52], who reported that the activities of total antioxidant capacity SOD and CAT showed a declining trend with increasing dietary FO replacement level, indicating that increased blended vegetable oil levels in the diets may have reduced the oxidation level in the hemolymph of the crabs.

### 4.5. Histomorphology of Liver

The results show the increase in lipid accumulation and hepatic adipose infiltration in the liver of fish fed the 6F0C, 2F4C, and 0F6C diets compared to those fed 5F1C and 4F2C diets. Several reports have shown that lipid infiltration in cell vacuoles becomes pronounced as vegetable oil replaces FO. We opinion that feeding with alternative plant oil sources did not impair the liver and intestine tissues probably by alleviating the role of ROS via improved antioxidative and proinflammatory responses [53]. However, in the present study, it did not damage the cells. Hepato- and viscera somatic indices represent the proportion of liver and viscera weights to whole-body weight, respectively, and can be used as indices of the nutritional status of red seabream [54] (FM: 28%) because the liver and viscera are energy storage organs. Results showed no significant changes in the HSI and VSI between red seabream groups, indicating that CO did not cause liver impairment. Previous studies on FO replacement using alternative lipid sources have reflected histomorphology alterations in tissues. For instance, supranuclear accumulation of lipid droplets was observed in the intestinal cells of some of the groups fed diets supplemented with vegetable oils (FM: 30%) [55]. Similarly, livers from these groups showed large amounts of lipid droplets within the hepatocytes. In contrast, Bell, et al. [56] reported a high degree of vacuolization due to lipid deposition in the livers of turbot fed marine FO (FM: 40%). This was not observed in fish fed diets containing borage oil. Accumulation of lipid droplets in enterocytes from the pyloric caeca and midgut has been observed in Arctic charr fed linseed oil [57]. Tissue histopathological interpretation of ultramphological alterations is difficult but can provide basic information for a thorough understanding of the metabolism of nutrients, including fatty acids, in various lipid sources.

## 5. Conclusions

The results of the present study suggest that FO can be replaced with CO in the feeds of farmed red seabream without compromising growth, blood chemistry, digestibility, and overall histological morphology. It is important to establish that alternative dietary lipids to FO are not only supplied in the correct proportions and balance for optimal growth and feed conversion but can maintain optimal health function and sustain overall biochemical and physiological responses. The present study shows that normal growth and overall health can be more successfully attained if dietary FO is replaced by CO, which provides a more physiologically balanced biochemical composition. In the future, bimolecular studies would provide interesting information on the mechanisms for the utilization of CO in the diets of marine species.

## Figures and Tables

**Table 1:** Experimental diets formulation and proximate composition.

Ingredient, g/kg DM	Test Diets				
	6F0C	5F1C	4F2C	2F4C	0F6C
Brown fish meal ^a^	460	460	460	460	460
Soybean meal ^b^	205	205	205	205	205
Fish oil ^c^	60	50	40	20	-
Camelina oil ^e^	-	10	20	40	60
Soybean Lecithin ^d^	30	30	30	30	30
EPA ^f^	50	50	50	50	50
DHA ^g^	50	50	50	50	50
Dextrin ^h^	50	50	50	50	50
Activated gluten ^i^	80	80	80	80	80
Mineral mix ^j^	40	40	40	40	40
Vitamin mix ^k^	40	40	40	40	40
Stay C ^l^	0.8	0.8	0.8	0.8	0.8
α-Cellulose ^m^	24.5	24.5	24.5	24.5	24.5
**Proximate composition**					
Crude Protein	498 ± 3	513 ± 4	508 ± 5	506 ± 0	508 ± 9
Crude Lipid	150 ± 9	144 ± 8	143 ± 13	135 ± 2	144 ± 10
Moisture	101 ± 1	93 ± 1	100 ± 4	90 ± 00	91 ± 5
Ash	101 ± 1	106 ± 00	101 ± 3	104 ± 3	103 ± 5
Energy (kJ/g) ^n^	2580 ± 6	2612 ± 14	2603 ± 14	2590 ± 4	2591 ± 2

^a^ Defatted brown fish meal. ^b^ J-OIL MILLS, Inc, Tokyo, Japan ^c^ Riken Vitamin, Tokyo, Japan. ^d^ Kanto Chemical Co., Inc. Tokyo. ^e^ Biopure oil, Box 194, Fort Qu Appele, SK SOG ISO. ^f^ Riken Vitamin, Tokyo, Japan. ^g^ Riken Vitamin, Tokyo, Japan. ^h,i^ Glico Nutrition Company Ltd. Osaka, Japan. Commercial name “A-glu SS. ^j^ Mineral mixture (mg/kg diet): MgSO_4_ (5.07), Na_2_HPO_4_ (3.23), K_2_HPO_4_ (8.87), Fe citrate (1.1), Ca lactate (12.09), Al(OH)_3_ (0.01), ZnSO_4_ (0.13), MnSO_4_ (0.03), Ca(IO_3_)_2_ (0.01), and CoSO_4_ (0.04). ^k^ Vitamin mixture (mg/kg diet): β-carotene (0.10), vitamin D_3_ (0.01), menadione NaHSO_3_·3H_2_O (K_3_) (0.05), dl-α-tocopherol acetate (E) (0.38), thiamine-nitrate (B_1_) (0.06), riboflavin (B_2_) (0.19), pyridoxine-HCl (B_6_) (0.05), cyanocobalamin (B_12_) (0.0001), biotin (0.01), inositol (3.85), niacin (nicotinic acid) (0.77), Ca pantothenate (0.27), folic acid (0.01), choline chloride (7.87), p-amino benzoic acid (0.38), and cellulose (1.92). ^l^ Stay-C: L-ascorbyl-2-monophosphate-Na/Ca (DSM Nutrition Japan K. K.). ^m^ Nippon Paper Chemicals, Tokyo, Japan. ^n^ Calculated using combustion values for protein, lipid, and carbohydrate of 23.6, 39.5, and 17.2 kJ/g.

**Table 2:** Fatty acid composition (mg/g lipids) in experimental diets.

Fatty Acid Type	Test Diet				
	6F0C	5F1C	4F2C	2F4C	0F6C
14:0	14.0 ± 1.8	13.8 ± 0.6	14.3 ± 0.3	11.2 ± 0.2	7.2 ± 0.2
16:0	51.1 ± 0.9	64.7 ± 0.6	86.0 ± 3.0	95.8 ± 50.0	114.0 ± 14.5
18:0	82.0 ± 2.0	35.0 ± 0.7	43.9 ± 3.9	37.4 ± 0.5	23.8 ± 4.3
**∑Saturated**	147.1 ± 3.6	132.5 ± 1.4	144.2 ± 2.1	144.4 ± 5.4	145 ± 4.1
16:1n-9	55 ± 1.8	50.8 ± 0.6	54.2 ± 0.7	32.9 ± 1.5	31.1 ± 0.1
18:1n-5	1.7 ± 0.5	1.1 ± 0.9	1.7 ± 0.1	2.7 ± 0.2	2.2 ± 0.6
18:1n-9	64.3 ± 1.2	86.1 ± 3.1	120.4 ± 2.0	137.1 ± 1.3	155.2 ± 2.6
20:1n-9	20.8 ± 4.0	17.0 ± 3.5	20.5 ± 1.3	11.0 ± 0.1	9.5 ± 0.3
22:1n-9	33.5 ± 3.5	17.8 ± 0.2	8.9 ± 0.4	7.9 ± 1.6	2.7 ± 1.5
** ∑ ** **MUFA**	175.5 ± 0.9	172.8 ± 5.8	205.7 ± 4.1	191.6 ± 6.5	200.7 ± 2.8
18:2n-6	41.1 ± 3.5^a^	52.7 ± 4.1	72.5 ± 3.6	84.8 ± 0.	103.4 ± 11.0
18:3n-6	6.4 ± 0.2	2.2 ± 0.1	1.6 ± 0.2	1.3 ± 0.1	0.4 ± 0.0
20:4n-6	8.2 ± 6.2	7.8 ± 4.6	6.4 ± 0.4	3.8 ± 0.2	2.7 ± 0.5
22:4n-6	2.1 ± 0.2	1.7 ± 0.1	1.4 ± 0.1	0.5 ± 0.0	0.0 ± 0.0
** ∑ n-6 fatty acids **	57.8 ± 8.3	64.4 ± 2.5	81.9 ± 7.6	90.4 ± 1.6	106.5 ± 1.6
18:3n-3	8.7 ± 1.6	10.0 ± 0.0	10.7 ± 0.7	12.3 ± 0.1	14.1 ± 8.8
18:4n-3	6.5 ± 0.2	3.1 ± 0.1	2.2 ± 0.1	1.8 ± 0.3	0.0 ± 0.0
20:3n-3	2.4 ± 0.5	1.4 ± 0.1	1.2 ± 0.1	0.5 ± 0.5	0.2 ± 0.2
20:4n-3	10.5 ± 0.2	6.5 ± 0.1	3.3 ± 0.3	3.5 ± 0.1	1.5 ± 0.4
20:5n-3	58.1 ± 20.2	57.0 ± 20.0	31.0 ± 2.0	21.3 ± 1.8	17.1 ± 1.8
22:5n-3	18.5 ± 4.5	14.2 ± 1.0	10.2 ± 0.7	9.0 ± 1.9	7.3 ± 2.0
22:6n-3	144.9 ± 23.9	129.0 ± 30.0	115.7 ± 11.1	101.0 ± 1.5	107.5 ± 2.5
** ∑ ** ** n-3 fatty acids **	249.6 ± 0.2	221.2 ± 2.6	174.3 ± 6.1	149.4 ± 2.3	146.5 ± 0.5
** ∑P UFA **	307.4 ± 12.4	285.6 ± 21.0	256.2 ± 19.2	239.8 ± 21.7	253. ± 46.2
** ∑-3HUFA **	234.4 ± 1.7	208.1 ± 16.2	161.4 ± 8.7	139.3 ± 0.4	133.6 ± 0.4
** ∑ n-3/n-6 ratio **	4.3	3.4	2.1	1.7	1.4
** ∑ EPA+DHA **	203 ± 21.1	186.1 ± 11.4	146.7 ± 2.1	122.3 ± 2.5	124.6 ± 1.2

Values are expressed as mean ± standard error (n = 2). Absence of superscript letters refers to non-significant differences between treatments (*p* > 0.05). Total PUFA is expressed as the sum of total n-3 fatty acids and total n-6 fatty acids. Total n-3HUFA is expressed as the sum of n-3 fatty acids in carbons of more than 20. The n-3:n-6 ratio is expressed as total n-3 PUFA divided by total n-6 PUFA. The sum of eicosapentaenoic acid (EPA; C20:5n-3) and docosahexaenoic acid (DHA; C22:6n-3) are essential fatty acids.

**Table 3:** Performance variables and biometric indices of red seabream (*Pagrus major*) fed the test diets for 56 days.

Parameters	Test Diets				
	6F0C	5F1C	4F2C	2F4C	0F6C
BW_0_ (g/fish)	1.8	1.9	1.8	1.9	1.9
BW_56d_ (g/fish)	26.2 ± 0.1	27.1 ± 0.2	27.3 ± 0.6	28.3 ± 0.1	26.5 ± 0.1
BWG (%)	1306.2 ± 47.9	1326.3 ± 6.1	1364.4 ± 27.2	1365.3 ± 49.1	1275.5 ± 25.4
SGR	4.7 ± 0.1	4.7 ± 0.0	4.7 ± 0.0	4.7 ± 0.1	4.6 ± 0.0
FI	25.6 ± 0.2	26.7 ± 0.4	27.8 ± 1.2	27.0 ± 0.8	27.2 ± 1.1
FCR	1.1 ± 0.0	1.1 ± 0.0	1.1 ± 0.1	1.0 ± 0.0	1.1 ± 0.0
HSI	1.1 ± 0.1	1.0 ± 0.1	0.9 ± 0.1	1.1 ± 0.4	1.1 ± 0.1
VSI	9.1 ± 0.7	9.5 ± 0.3	7.5 ± 0.6	8.5 ± 0.7	8.9 ± 0.7
CF	2.3 ± 0.0	2.3 ± 0.1	2.4 ± 0.1	2.2 ± 0.0	2.5 ± 0.2
SR	82.2 ± 5.8	97.7 ± 2.2	88.8 ± 8.0	100 ± 0.0	86.6 ± 6.62

Values are expressed as the mean ± standard error (n = 3). BW_56d_ = body weight at 56 day; BWG = body weight gain; SGR = specific growth rate; FI = feed intake; FCR = feed conversion ratio; HSI = hepatosomatic index; VSI = viscerasomatic index; CF = condition factor; SR = survival rate (%).

**Table 4:** Carcass proximate analysis (g/kg, dry matter basis) of red seabream (*Pagrus major*) fed the experimental diets for 56-days.

Parameter	Test Diets				
	FO_6_CO_0_	FO_5_CO_1_	FO_4_CO_2_	FO_2_CO_4_	FO_0_CO_6_
Moisture	704 ± 4.8	694 ± 4.5	702 ± 11.8	697 ± 6.9	700 ± 4.7
Crude Protein	546 ± 6	512 ± 00	544 ± 45	539 ± 4	531 ± 2
Crude Lipid	291 ± 3 ^a^	232 ± 1 ^b^	212 ± 4 ^bc^	232 ± 1 ^b^	244 ± 9 ^b^
Ash	157 ± 2	154 ± 3	161 ± 2	155 ± 1	155 ± 1

Values are expressed as the mean ± standard error (n = 3). Absence of superscript letters refers to non-significant differences between treatments (*p* > 0.05) and presence of different superscript letters refers to significant differences between treatments (*p* < 0.05).

**Table 5:** Fatty acid composition (mg/g lipid) in the liver of red seabream (*Pagrus major*) fed the experimental diets 56-days.

Fatty Acid Type	Test Diet				
	6F0C	5F1C	4F2C	2F4C	0F6C
14:0	27.1 ± 0.3	29.1 ± 0.2	33.5 ± 0.6	32.0 ± 0.1	26.1 ± 0.2
16:0	123.2 ± 0.1 ^a^	109.5 ± 0.1 ^a^	102.5 ± 0.7 ^b^	96.0 ± 0.3 ^b^	85.9 ± 0.1 ^b^
18:0	67.5 ± 0.0 ^a^	57.5 ± 0.2 ^a^	46.1 ± 0.6 ^ab^	36.0 ± 0.6 ^bc^	26.3 ± 0.5 ^bc^
**∑Saturated**	217.8 ± 0.0 ^a^	195.6 ± 0.3 ^a^	182.1 ± 0.3 ^ab^	164.0 ± 0.9 ^b^	138.3 ± 0.1 ^bc^
16:1n-9	61.5 ± 0.5 ^a^	44.8 ± 0.2 ^a^	37.7 ± 0.9 ^a^	32.9 ± 0.7 ^ab^	27.0 ± 0.7 ^b^
18:1n-5	4.9 ± 0.2 ^a^	2.0 ± 0.1 ^a^	3.0 ± 0.1 ^a^	1.7 ± 0.01 ^b^	0.0 ± 0.7 ^c^
18:1n-9	70.1 ± 0.01 ^a^	98.3 ± 0.5 ^b^	124.6 ± 0.8 ^c^	148.4 ± 0.1 ^c^	168.6 ± 1.0 ^c^
20:1n-9	51.0 ± 0.2 ^a^	44.2 ± 0.2 ^a^	36.8 ± 0.5 ^b^	37.5 ± 0.0 ^b^	31.2 ± 2.1 ^c^
22:1n-9	34.5 ± 0.3 ^a^	31.0 ± 0.01 ^a^	23.0 ± 0.0 ^a^	20.6 ± 0.5 ^ab^	13.6 ± 0.1 ^b^
∑MUFA	222.0 ± 0.6	219.5 ± 0.1	223.1 ± 0.2	241.1 ± 0.6	240.2 ± 2.1
18:2n-6	84.0 ± 0.1	86.1 ± 0.2	99.2 ± 2.7	108.9 ± 1.5	113.2 ± 4.6
18:3n-6	5.8 ± 0.2 ^a^	3.7 ± 0.1 ^a^	3.1 ± 0.2 ^a^	2.5 ± 0.8 ^ab^	1.5 ± 0.0 ^b^
20:4n-6	9.6 ± 0.5 ^a^	5.3 ± 0.6 ^ab^	3.6 ± 0.0 ^b^	3.0 ± 0.0 ^b^	3.2 ± 0.0 ^b^
**∑n-6 fatty acids**	99.6 ± 0.2	95.1 ± 0.0	105.3 ± 0.2	114.4 ± 1.3	117.7 ± 3.2
18:3n-3	15.5 ± 0.4 ^a^	18.6 ± 0.4 ^ab^	20.2 ± 0.6 ^b^	25.1 ± 2.1 ^bc^	27.9 ± 0.0 ^bc^
18:4n-3	12.5 ± 0.1 ^a^	9.1 ± 0.2 ^ab^	9.6 ± 0.2 ^ab^	7.9 ± 0.0 ^b^	3.9 ± 0.0 ^c^
20:3n-3	7.4 ± 0.3 ^a^	3.6 ± 0.1 ^b^	1.7 ± 0.0 ^bc^	0.6 ± 0.0 ^c^	0.9 ± 0.4 ^c^
20:4n-3	6.3 ± 0.5 ^a^	3.7 ± 0.02 ^b^	2.3 ± 0.01 ^b^	2.6 ± 0.00 ^b^	0.0 ± 0.0 ^c^
20:5n-3	80.5 ± 1.6 ^a^	59.6 ± 0.2 ^b^	55.7 ± 0.3 ^b^	47.4 ± 0.8 ^b^	52.5 ± 2.4 ^b^
22:5n-3	23.0 ± 0.6 ^a^	14.5.0 ± 0.2 ^b^	7.1 ± 0.5 ^c^	6.7 ± 2.0 ^c^	6.5 ± 0.1 ^c^
22:6n-3	159.0 ± 0.1	145 ± 0.8	142.2 ± 0.5	128.4 ± 0.7	120.2 ± 0.0
**∑n-3 fatty acids**	304.2 ± 2.6 ^a^	254.9 ± 2.9 ^ab^	238.8 ± 0.0 ^ab^	218.7 ± 0.9 ^ab^	211.9 ± 2.1 ^ab^
**∑PUFA ^1^**	403.8 ± 0.9	350 ± 5.3	344.1 ± 0.0	333.1 ± 1.1	329.6 ± 0
**∑-3HUFA ^2^**	276.2 ± 5.9	226.4 ± 2.5	209.0 ± 2.9	185.7 ± 4.30	180.1 ± 5.2
**∑n-3/n-6 ratio ^3^**	3.1	2.7	2.3	2.0	1.8
**∑EPA + DHA ^4^**	239.5 ± 0.1 ^a^	204.6 ± 0.0 ^a^	197.9 ± 0.0 ^a^	175.8 ± 0.0 ^ab^	172.7 ± 0.3 ^ab^

Values are expressed as mean ± standard error (n = 2). Absence of superscript letters refers to non-significant differences between treatments (*p* > 0.05) and presence of different superscript letters refers to significant differences between treatments (*p* < 0.05). ^1^ Total PUFA is expressed as the sum of total n-3 fatty acids and total n-6 fatty acids. ^2^ Total n-3HUFA is expressed as the sum of n-3 fatty acids in carbons of more than 20. ^3^ The n-3: n-6 ratio is expressed as total n-3 PUFA divided by total n-6 PUFA. ^4^ Essential fatty acids = the sum of eicosapentaenoic acid (EPA; C20:5n-3) and docosahexaenoic acid (DHA; C22:6n-3).

**Table 6:** Fatty acid composition (mg/g lipid) in the muscle of red seabream (*Pagrus major*) fed the experimental diets for 56-days.

Fatty Acid Type	Test Diet				
	6F0C	5F1C	4F2C	2F4C	0F6C
14:0	26.8 ± 0.1 ^a^	17.1 ± 0.2 ^a^	12.4 ± 0.2 ^ab^	11.6 ± 0.4 ^abc^	8.6 ± 0.1 ^c^
16:0	73.0 ± 0.2	75.2 ± 0.1	82.4 ± 0.7	99.6 ± 0.3	108.2 ± 0.1
18:0	96.3 ± 0.0 ^a^	53.2 ± 0.2 ^ab^	45.5 ± 0.6 ^ab^	30.1 ± 0.6 ^bc^	27.9 ± 0.5 ^bc^
**∑Saturated**	196.1 ± 5.6	145.5 ± 0.3	140.3 ± 0.3	141.3.4 ± 0.9	144.7 ± 0.1
16:1n-9	83.6 ± 0.4 ^a^	65.7 ± 0.2 ^ab^	44.0 ± 0.9 ^ab^	31.4 ± 0.7 ^ab^	29.9 ± 0.5 ^b^
18:1n-5	1.4 ± 0.2	0.2 ± 0.1	0.2 ± 0.1	- ± -	- ± -
18:1n-9	76.6 ± 2.5 ^a^	101.3 ± 0.5 ^a^	126.2 ± 0.8 ^ab^	145.8 ± 0.1 ^ab^	164.2 ± 1.0 ^b^
20:1n-9	21.2 ± 0.0 ^a^	13.5 ± 0.2 ^b^	14.2 ± 0.5 ^b^	13.6 ± 0.0 ^b^	11.0 ± 1.2 ^b^
22:1n-9	35.5 ± 0.5	26.0 ± 0.01	23.5 ± 0.0	20.3 ± 0.5	20.9 ± 0.1
** ∑ ** **MUFA**	218.3 ± 1.6	206.7 ± 0.1	208.1 ± 0.2	211.1 ± 0.6	226 ± 2.1
18:2n-6	63.2 ± 0.1 ^a^	80.9 ± 0.2 ^ab^	118.4 ± 2.7 ^ab^	128.8 ± 1.5 ^ab^	144.0 ± 1.3 ^b^
18:3n-6	6.1 ± 0.2 ^a^	1.7 ± 0.1 ^b^	0.1 ± 0.2 ^b^	0.9 ± 0.8 ^b^	0.0 ± 0.0 ^c^
20:4n-6	8.6 ± 0.5 ^a^	6.2 ± 0.6 ^a^	4.3 ± 0.0 ^a^	1.0 ± 0.0 ^b^	0.0 ± 0.0 ^c^
** ∑ n-6 fatty acids **	77.9 ± 0.2	88.8 ± 2.1	122.8 ± 0.2	130.7 ± 1.3	144.0 ± 3.2
18:3n-3	10.6 ± 0	10.1 ± 0.4	12.4 ± 0.6	13.7 ± 2.1	14.7 ± 0.0
18:4n-3	4.1 ± 0.02 ^a^	1.3 ± 0.2 ^b^	1.2 ± 0.2 ^b^	1.2 ± 0.0 ^b^	1.2 ± 0.0 ^b^
20:3n-3	4.5 ± 0.2 ^a^	1.2 ± 0.1 ^b^	0.6 ± 0.0 ^c^	0.6 ± 0.0 ^c^	1.1 ± 0 ^b^
20:4n-3	5.3 ± 0.3 ^a^	2.1 ± 0.02 ^b^	2.2 ± 0.2 ^b^	2.2 ± 0.0 ^b^	0.0 ± 0.0 ^c^
20:5n-3	64.4 ± 1.1 ^a^	60.0 ± 1.6 ^a^	24.0 ± 3.1 ^b^	24.9 ± 0.8 ^b^	19.8 ± 0.8 ^b^
22:5n-3	4.0 ± 0.0 ^a^	0.2 ± 0.2 ^b^	0.1 ± 0.5 ^b^	0.3 ± 0.03 ^b^	0.1 ± 0.01 ^b^
22:6n-3	147.2 ± 5.3	133.7 ± 2.8	123.3 ± 1.5	112.4 ± 7	100.0 ± 0.0
** ∑ ** ** n-3 fatty acids **	237.8 ± 2.6	209.6 ± 1.7	161.8 ± 3.0	152.3 ± 1.1	131.9 ± 3.1
** ∑P UFA ^1^**	315.7 ± 2.1	298.4 ± 5.3	284.6 ± 0.0	283.3 ± 1.1	275 ± 2.3
** ∑-3HUFA ^2^**	225.4 ± 0.5	197 ± 2.5	150.2 ± 2.9	140.4 ± 4.30	121.0 ± 5.2
** ∑ n-3/n-6 ratio ^3^**	3.0	2.3	1.3	1.2	1.0
** ∑ EPA + DHA ^4^**	211.6 ± 1.3 ^a^	193.7 ± 0.0 ^a^	147.3 ± 0.0 ^a^	137.3 ± 0.0 ^ab^	119.8 ± 0.3 ^b^

Values are expressed as mean ± standard error (n = 2). Absence of superscript letters refers to non-significant differences between treatments (*p* > 0.05) and presence of different superscript letters refers to significant differences between treatments (*p* < 0.05). ^1^ Total PUFA is expressed as the sum of total n-3 fatty acids and total n-6 fatty acids. ^2^ Total n-3HUFA is expressed as the sum of n-3 fatty acids in carbons of more than 20. ^3^ The n-3: n-6 ratio is expressed as total n-3 PUFA divided by total n-6 PUFA. ^4^ The sum of eicosapentaenoic acid (C20:5n-3) and docosahexaenoic acid (C22: 6n-3) are essential fatty acids.

**Table 7:** Apparent digestibility (%) of fatty acids.

Fatty Acid Type	Test Diet				
	6F0C	5F1C	4F2C	2F4C	0F6C
14:0	91.2 ± 3	92.5 ± 2	94.1 ± 6	96.0 ± 1	96.6 ± 2
16:0	94.3 ± 1	86.4 ± 1	91.7 ± 07	92.3 ± 3	90.7 ± 1
18:0	95.3 ± 0	85.1 ± 2	95.2 ± 6	88.6 ± 6	85.5 ± 5
**∑Saturated**	93.6 ± 0	88.0 ± 3	93.6 ± 3	92.3 ± 9	90.9 ± 1
16:1n-9	93.5 ± 1	93.7 ± 1	98.2 ± 1	96.9 ± 13	94.1 ± 0.1
18:1n-5	95.0 ± 2	97.5 ± 1	92.3 ± 1	97.7 ± 0.1	95.9 ± 7
18:1n-9	92.7 ± 0.1	89.6 ± 5	98.2 ± 8	96.8 ± 1	95.1 ± 1
20:1n-9	92.1 ± 2	92.1 ± 2	98.2 ± 5	96.1 ± 0	94.6 ± 21
22:1n-9	99.1 ± 2	99.4 ± 1	98.2 ± 2	95.3 ± 2	97.0 ± 1
**∑MUFA**	94.5 ± 6	94.5 ± 1	97.0 ± 2	96.6 ± 6	95.3 ± 21
18:2n-6	93.6 ± 3	84.4 ± 3	98.5 ± 6	97.3 ± 3	96.1 ± 1
18:3n-6	94.1 ± 2	97.5 ± 4	94.6 ± 0.1	97.7 ± 2	96.3 ± 0
20:4n-6	93.6 ± 5	83.0 ± 6	99.0 ± 0	97.0 ± 0	96.0 ± 00
22:4n-6	94.2 ± 0.1	95.5 ± 1.0	98.6 ± 0.1	97.6 ± 0.0	94.7 ± 0.2
** ∑n-6 fatty acids **	93.9 ± 0.1	90.1 ± 2.1	97.7 ± 0.2	97.4 ± 2.6	95.8 ± 0.3
18:3n-3	95.1 ± 0.6	81.2 ± 0.9	99.2 ± 0.1	98.5 ± 4.1	97.9 ± 0.0
18:4n-3	95.5 ± 0.01	97.0 ± 0.2	99.4 ± 0.0	99.0 ± 0.0	97.1 ± 0.0
20:3n-3	95.3 ± 0.2	88.6 ± 0.8	99.4 ± 0.5	98.8 ± 2.0	96.7 ± 0.3
20:4n-3	95.1 ± 0.01	94.4 ± 0.1	98.6 ± 0.3	98.6 ± 0.1	97.4 ± 0.5
20:5n-3	95.7 ± 0.3	95.2 ± 0.5	98.4 ± 0.4	98.5 ± 0.1	97.8 ± 0.1
22:5n-3	98.7 ± 0.1	94.8 ± 0.7	99.8 ± 0.1	98.9 ± 0.3	97.2 ± 0.4
22:6n-3	94.6 ± 0.2	93.5 ± 1.5	99.6 ± 0.0	97.6 ± 0.9	96.6 ± 0.1
** ∑n-3 fatty acids **	95.7 ± 0.01	92.1 ± 1.7	99.2 ± 0.0	98.6 ± 0.5	97.2 ± 0.4
** ∑P UFA ^1^**	94.8 ± 0.2	91.1 ± 3.0	98.4 ± 0.0	98.0 ± 0.2	96.5 ± 0.4
** ∑-3HUFA ^2^**	95.9 ± 0.4	93.3 ± 0.1	99.2 ± 0.04	98.5 ± 0.2	97.1 ± 0.1
** ∑ n-3/n-6 ratio ^3^**	1.02	1.02	1.01	1.0	1.04
** ∑ EPA + DHA ^4^**	95.2 ± 0.2	94.3 ± 0.1	99.0 ± 0.2	98.0 ± 0.1	97.2 ± 0.2

Values are expressed as mean ± standard error (n = 2). Absence of superscript letters refers to non-significant differences between treatments (*p* > 0.05). ^1^ Total PUFA is expressed as the sum of total n-3 fatty acids and total n-6 fatty acids. ^2^ Total n-3HUFA is expressed as the sum of n-3 fatty acids in carbons of more than 20. ^3^ The n-3: n-6 ratio is expressed as total n-3 PUFA divided by total n-6 PUFA. ^4^ The sum of eicosapentaenoic acid (C20:5n-3) and docosahexaenoic acid (C22: 6n-3) are essential fatty acids.

**Table 8:** Blood health of red seabream (*Pagrus major*) fed the experimental diets for 56-days.

Parameters	Test Diets				
	6F0C	5F1C	4F2C	2F4C	0F6C
Haematocrit (%)	36.0 ± 1.1	37.3 ± 3.1	36.3 ± 3.1	36.6 ± 1.2	33.0 ± 4.2
Glucose (mg/dL)	72.3 ± 4.0	71.3 ± 14.3	78.3 ± 10.5	72.3 ± 19.0	88 ± 5.5
Serum total protein (g/dL)	3.1 ± 0.1	3.4 ± 0.2	3.8 ± 0.4	3.3 ± 0.0	3.4 ± 0.2
Total Cholesterol (mg/dL)	213.6 ± 8.9	238.0 ± 24.2	241.6 ± 6.9	196.0 ± 8.0	229.6 ± 21.6
BUN (mg/dL)	11.3 ± 2.0	8.3 ± 2.0	13.0 ± 3.2	14.6 ± 1.2	9.0 ± 1.1
T-Bil (mg/dL)	0.4 ± 0.1	0.4 ± 0.1	0.4 ± 0.0	0.5 ± 0.0	0.5 ± 0.0
Triglyceride (mg/dL)	393.3 ± 62.6	476.6 ± 23.3	437.6 ± 62.3	415.0 ± 58.0	497.6 ± 2.3
ALT (UI/L)	52.3 ± 11.3	65.5 ± 15.4	64.3 ± 7.6	50 ± 1.3	53.0 ± 3
AST (UI/L)	43.0 ± 10.5	44.0 ± 29.1	38.0 ± 4.8	42.3 ± 9.5	69.3 ± 16.3

Data represent the mean ± SEM (n = 3). Absence of letters indicates no significant difference between treatments (*p* > 0.05). Alanine aminotransferase test; ALT, aspartate aminotransferase test; AST, blood urea nitrogen; BUN, total bilirubin, T-Bil.

## Data Availability

The datasets generated during and analysed during the current study are available from the corresponding author on reasonable request.
